# The Efficacy of Device Designs (Mono-block or Bi-block) in Oral Appliance Therapy for Obstructive Sleep Apnea Patients: A Systematic Review and Meta-Analysis

**DOI:** 10.3390/ijerph16173182

**Published:** 2019-08-31

**Authors:** Hiroyuki Ishiyama, Daichi Hasebe, Kazumichi Sato, Yuki Sakamoto, Akifumi Furuhashi, Eri Komori, Hidemichi Yuasa

**Affiliations:** 1 Orofacial Pain Management, Graduate School of Medical and Dental Sciences, Tokyo Medical and Dental University (TMDU), 1-5-45 Yushima Bunkyo-ku, Tokyo 1138510, Japan; 2 Division of Reconstructive Surgery for Oral and Maxillofacial Region, Department of Tissue Regeneration and Reconstruction, Niigata University Graduate School of Medical and Dental Sciences, 2-5274 Gakkocho-Dori, Cyuo-ku, Nigata-shi, Nigata 9518514, Japan; 3 Department of Oral Medicine, Oral and Maxillofacial Surgery, Tokyo Dental College, 5-11-13 Sugano Ichikawa-shi, Chiba 2728513, Japan; 4 Department of Oral Surgery, Hironokogen Hospital, 3-1-1 Kitayamadai Nishi-ku Kobe-shi, Hyogo 6512215, Japan; 5 Department of Oral and Maxillofacial Surgery, Aichi Medical University, 1-1 Yazakokarimata Nagakute-shi, Aichi 4801103, Japan; 6 Division of Medicine for Function and Morphology of Sensor Organ, Dentistry and Oral Surgery, Osaka Medical College, 2-7 Daigaku-machi Takatsuki-shi, Osaka 5698686, Japan; 7 Department of Oral and Maxillofacial Surgery, National Hospital Organization Toyohashi Medical Center, 50 Imure-chou Aza Hamamichi-Ue, Toyohashi-shi, Aichi 4408510, Japan

**Keywords:** obstructive sleep apnea, oral appliance, systematic review, mono-block, bi-block

## Abstract

Oral appliance (OA_m_) therapy has demonstrated efficacy in treating obstructive sleep apnea (OSA). The aim of this systematic review was to clarify the efficacy of device designs (Mono-block or Bi-block) in OA_m_ therapy for OSA patients. We performed a meta-analysis using the Grading of Recommendations, Assessment, Development, and Evaluation (GRADE) system. Two studies (Mono-block OA_m_ versus Bi-block OA_m_) remained eligible after applying the exclusion criteria. When comparing Mono-block OA_m_ and Bi-block OA_m_, Mono-block OA_m_ significantly reduced the apnea–hypopnea index (2.92; 95% confidence interval (95%CI), 1.26 to 4.58; *p =* 0.0006), and patient preference for Mono-block OA_m_ was significantly higher (2.06; 95%CI, 1.44 to 2.06; *p* < 0.0001). Lowest SpO_2_, arousal index, non-REM stage 3, sleep efficiency, Epworth Sleepiness Scale (ESS), Snoring Scale, and side effects were not significantly different between the two groups (lowest SpO_2_: −11.18; 95%CI, −26.90 to 4.54; *p* = 0.16, arousal index: 4.40; 95%CI, −6.00 to 14.80; *p* = 0.41, non-REM stage 3: −2.00; 95%CI, −6.00 to 14.80; *p* = 0.41, sleep efficiency: −1.42, 95%CI, −4.71 to 1.86; *p* = 0.40, ESS: 0.12; 95%CI, −1.55 to 1.79; *p* = 0.89, Snoring Scale: 0.55; 95%CI, −0.73 to 1.83, *p* = 0.55, side effects: 1.00, 95%CI, 0.62 to 1.61, *p* = 1.00). In this systematic review, the use of Mono-block OA_m_ was more effective than Bi-block OA_m_ for OSA patients.

## 1. Introduction

Obstructive sleep apnea (OSA) is a disease where the upper airway narrows or collapses repeatedly during sleep [1,2,3,4]. Anatomical factors of the upper airway and decompensation due to neuromodulation have been reported as the main causes of OSA [3,4]. In particular, obesity and micrognathia worsen the anatomical balance around the throat, increase pharyngeal obstruction, and are highly involved in the onset of OSA [5].

Oral appliance (OA) therapy is a treatment option for OSA [6]. Among the different appliances, mandibular advancement-type oral appliances (OA_m_) are mainly used. Wearing an OA_m_ will pull the lower jaw forward and cause an expansion of the upper airway and oral cavity volume, thus preventing upper airway obstruction during sleep [7]. OA_m_ are outstanding in their simplicity, economic efficiency, and portability, and recent reports show the beneficial effects of OA_m_ even for severe cases of OSA [8,9]. An American Academy of Sleep Medicine (AASM) and American Academy of Dental Sleep Medicine (AADSM) clinical practice guideline recommends that sleep physicians consider the prescription of oral appliances, rather than no treatment, for adult patients with OSA who are intolerant of continuous positive airway pressure (CPAP) therapy or prefer alternate therapy [10].

There are various types of OA_m_ devices [11]. However, most fall under one of two major designs: Mono-block and Bi-block types. As for Mono-block OA_m_, since both the upper and lower jaw are fixed in place by the same device, jaw movement is restricted, leading to a sense of constraint during sleep. Further, there are many cases in which the anteroposterior position of the lower jaw cannot be easily adjusted. On the other hand, the Bi-block OA_m_ has separate constructions for the upper and lower jaws and is equipped with connectors or attachments that advance the mandible. The lower jaw can be opened and moved sideways, resulting in a reduced sense of constraint and discomfort for the patient. The Bi-block OA_m_ has a device that completely separates the upper and lower jaws, and a movable device that cannot completely separate the upper and lower jaws. The main feature of the Bi-block OA_m_ is the ease of anteroposterior position adjustment of the lower jaw by means of a screw or connector built into the device. However, the treatment principle of expanding the upper airway by moving the lower jaw forward is the same in Mono-block OA_m_ and Bi-block OA_m_.

Although there are several systematic reviews (SRs) on OA_m_ designs, a decisive conclusion as to which design is most effective for OSA treatment has not yet been reached. Therefore, in this systematic review, we extracted research results from past comparative studies of Mono-block OA_m_ and Bi-block OA_m_ (or similar designs), conducted a meta-analysis, and evaluated each design according to its OSA treatment efficacy. If one type was superior to the other, we evaluated the different designs to figure out the most effective design of that type. Our meta-analysis was performed using the Grading of Recommendations Assessment, Development, and Evaluation (GRADE) system [12,13].

## 2. Materials and Methods 

This SR was performed following the PRISMA (Preferred Reporting Items for Systematic Reviews and Meta-Analyses) guidelines [14]. The protocol for this review was registered with the international prospective register of systematic reviews (PROSPERO) with the registration number CRD42019131303.

### 2.1. Eligibility Criteria

For this review, we referenced studies using OA_m_ on OSA patients aged 18 years and above. When selecting studies for this review, the following criteria regarding the OA_m_ had to be fulfilled: (1) the device was custom-made by creating a dental impression of the lower jaw for every patient, (2) the OA_m_ design was classified only as Mono-block and Bi-block types, and (3) titration of the OA_m_ (including adjustment) was performed. Further, in order to comprehensively evaluate the influence of the structure of the OA_m_, we did not establish any restrictions regarding the layout, material, thickness, or detailed design of OA_m_ (type of connector used in Bi-block, presence of air vents in Mono-block, etc.). Another criterion for the inclusion of studies was the evaluation of diagnostic and therapeutic OSA treatment efficacy through either polysomnography (PSG) or Out of Center Sleep Testing (OCST). We excluded studies that included patients under the age of 18 years and patients with other coexisting sleep disorders, studies in which ready-made devices were used, and studies in which results were achieved by securing the tongue in a forward position through tongue retaining devices.

### 2.2. Literature Search

**Figure 1:**
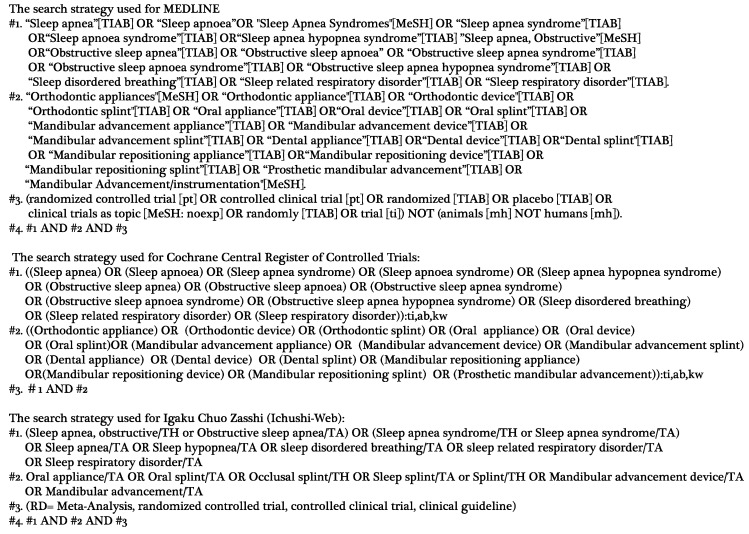
The search strategy used in this systematic review.

The searches were performed using the following databases: MEDLINE, Cochrane Central Register of Controlled Trials (CENTRAL), and Igaku Chuo Zasshi (Ichushi-Web). In the literature search, there were no restrictions on the year or language published. We selected only randomized controlled trials (RCTs) in the design of the OA_m_, detailing the comparison of the Mono-block to the Bi-block or similar designs. Prospective and retrospective non-randomized clinical studies, case reports/case series, conferences abstracts, and reviews were excluded. A search strategy is shown in Figure 1 and the final literature search was conducted and completed on 27 April 2019. When searching, if an unpublished article such as a paper on conference proceedings was found, we investigated whether or not it was published later.

### 2.3. Study Selection Procedure

Eligible studies were selected in two phases. In the first phase, two authors independently screened the titles and abstracts. In the second phase, the full texts of all potentially eligible studies identified during the first phase were independently reviewed by two authors. During the full-text assessment, irrelevant studies were excluded based on the inclusion and exclusion criteria. When the selection differed between the two authors, we added a third author and solved the issue by discussion.

### 2.4. Data Extraction

Data were extracted independently by three authors. The important information collected was the author name, year of publication, study design, type of device, number of subjects and dropouts, mean age, body mass index (BMI), mean baseline apnea-hypopnea index (AHI), follow-up periods, and study outcome. The extracted results included values for both before and after the OA_m_ treatment. The primary outcomes were mortality rates and cardiovascular events, and surrogate outcomes were as follows: (1) treatment effects: AHI (including the respiratory disturbance index (RDI) and respiratory event index (REI)), lowest SpO_2_, arousal Index, sleep efficiency, sleep stage (non-REM stage 3: NREM stage 3), subjective daytime sleepiness (the Epworth Sleepiness Scale (ESS)), the loudness and effects (disturbance factor) of snoring (the Snoring Scale (SS)); (2) adherence (the duration of OA_m_ usage at night and the number of days the OA_m_ was used in the preceding week); (3) sleep-related quality of life (SF-36 Physical Component, SF-36 Mental Component); (4) hypertension: systolic blood pressure, diastolic blood pressure; (5) side effects: temporomandibular disorders (arthralgia or myalgia), tooth pain, occlusal changes (overbite, overjet), the changes of occlusal contact, the changes of bite force; and (6) patient preference. If the standard error of the mean (SEM) was reported for outcomes, the standard deviation (SD) was also calculated from the number of subjects in the study and the reported SEM.

### 2.5. Data Synthesis and Statistical Analysis

Meta-analyses were conducted using Review Manager (Nordic Cochrane Centre, Cochrane Collaboration, 2014 Copenhagen, Denmark) version 5.3 software by pooling data across studies for each outcome measure. The available case analysis was applied where data were missing. When multiple studies were combined, the risk ratio (RR) and the mean difference (MD) were used. The effectiveness was evaluated based on RR or MD and its 95% confidence interval (CI) in each study. A forest plot was constructed using the RR or MD of the outcome variable in each study. All analyses were performed using the fixed-effects model with results displayed as a forest plot.

### 2.6. Quality Assessment of Included Studies

The evaluation of the quality of evidence in this SR was performed using the Grading of Recommendations Assessment, Development, and Evaluation (GRADE) process [12]. The GRADE classification was downgraded by one level for each of the five factors we considered, which were study limitations, inconsistency, indirectness, imprecision, and publication bias. Seven authors judged whether the five factors were present for each outcome. The risk of bias of the studies included for this review were evaluated using the Cochrane Risk of Bias tool described in the Cochrane Handbook for Systematic Reviews of Intervention. A GRADE evidence profile was created using the GRADEpro software for each clinical domain and adverse events. The following definitions of the quality of the evidence were applied [13]: high quality (further research is very unlikely to change our confidence in the estimate of the effect), moderate quality (further research is likely to have an important effect on our confidence in the estimate of the effect and may change the estimate), low quality (further research is very likely to have an important effect and is likely to change the estimate), very low quality (we are very uncertain about the estimate).

## 3. Results

### 3.1. Literature Search and Selection Results

**Figure 2:**
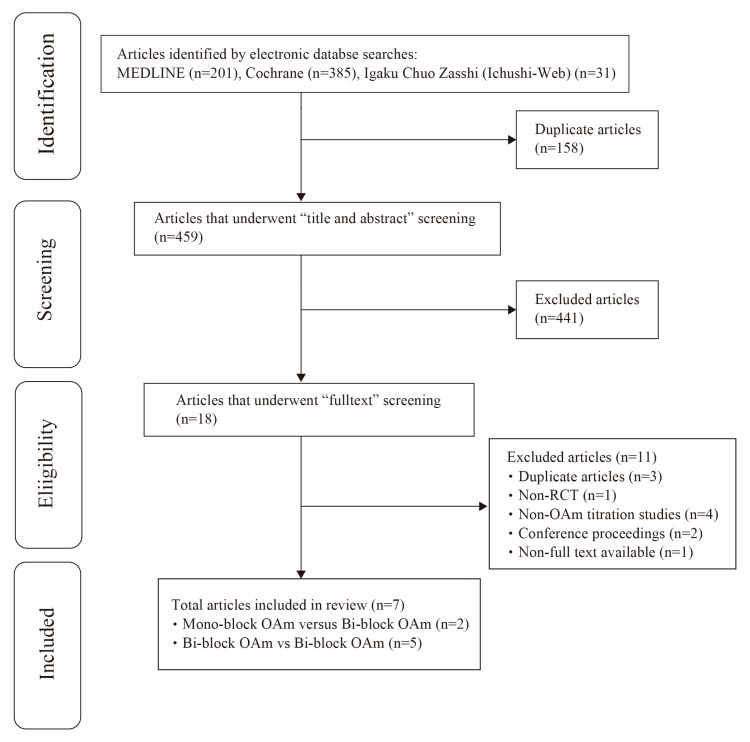
PRISMA flow diagram of selection process.

The PRISMA flow diagram is presented in Figure 2. Our selection of studies included 201 MEDLINE articles, 385 Cochrane Central Register of Controlled Trials (CENTRAL) articles, and 31 Igaku Chuo Zasshi (Ichushi-Web) articles. Excluding 158 duplicate articles, we screened the titles and abstracts of 459 articles. We then narrowed our selection down to 18 articles, of which 10 were excluded after confirming the eligibility of each articles (three duplicate articles, one non-RCT article, four articles without OA_m_ titration and with fixed mandibular position at the time of OA_m_ device fabrication, two cases of conference proceedings, one case in which the respective paper could not be located). Finally, seven articles were accepted (two articles of Mono-block OA_m_ versus Bi-block OA_m_ [15,16] and five articles of Bi-block OA_m_ versus Bi-block OA_m_ [17,18,19,20,21]). We were not able to find any articles covering a comparison among Mono-block OA_m_.

### 3.2. Key Features of the Included Studies

The main characteristics of the included studies are shown in Table 1 and Table 2. Of the seven [15,16,17,18,19,20,21] articles accepted, six were randomized crossover studies [15,16,17,18,19,20] and one was a randomized parallel group comparison test [21]. There were six short-term follow-ups [15,16,17,18,19,20] and one long-term follow-up [21].

**Figure 3:**
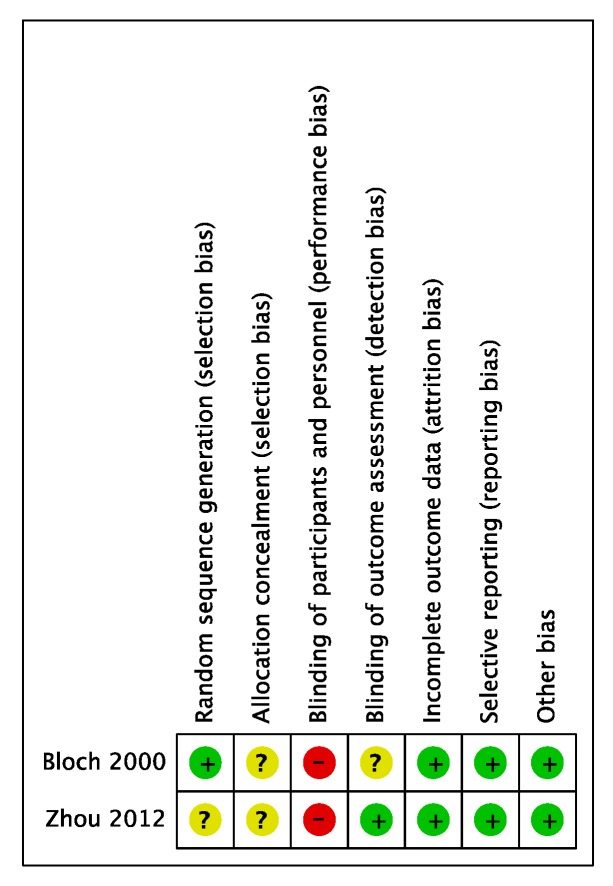
Assessment of risk of bias of the included studies (Mono-block OA_m_ versus Bi-block OA_m_) for systematic review.

**Figure 4:**
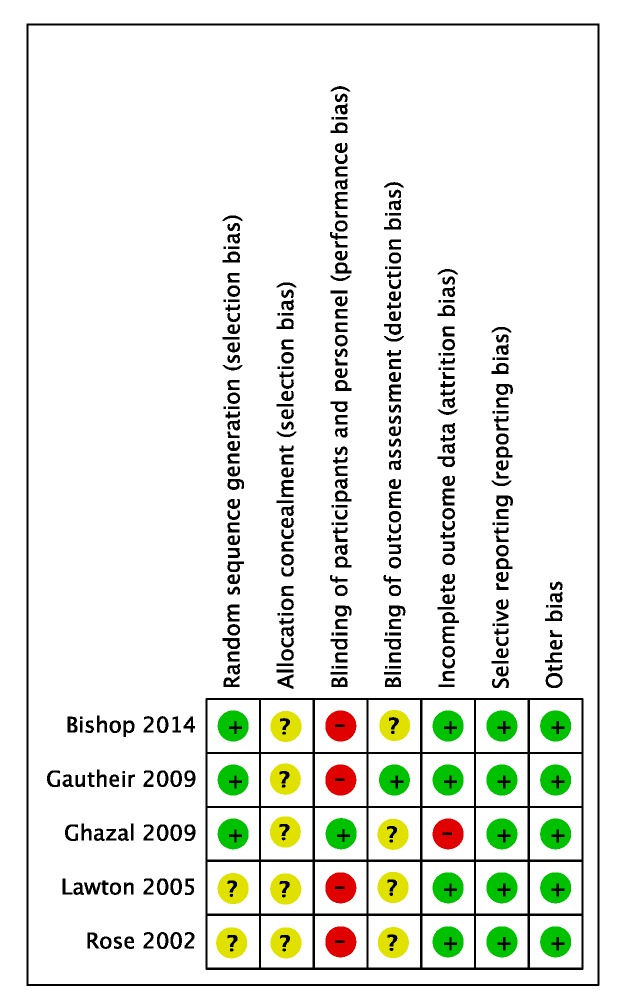
Assessment of risk of bias of the included studies (Bi-block OA_m_ versus Bi-block OA_m_) for systematic review.

A risk of bias summary of randomized studies in Mono-block OA_m_ versus Bi-block OA_m_ according to Cochran’s tool is shown in Figure 3. The study design adopted in two [15,16] of the selected studies was a randomized crossover study, which we regarded as having high risk of performance bias. However, in the outcome evaluation of both reports, the bias had been judged to be small and thus we evaluated it as low risk. Another potential bias was that subsidies were granted as research funds [15]. However, since these were received by the university, we evaluated the case as having low risk of bias. A risk of bias summary of randomized studies in Bi-block OA_m_ versus Bi-block OA_m_ according to Cochran’s tool is shown in Figure 4. The study design adopted in four out of five articles [17,18,19,20,21] was a randomized crossover study, which we regarded as having high risk of performance bias. Additionally, regarding attrition bias, a dropout was recorded in the middle of one study [20]. Since no ITT (intention-to-treat) analysis was conducted, we evaluated the case as having a high risk of case reduction bias. Another potential bias was that subsidies were granted as research funds [21]. However, since these were public funds, we evaluated the case as having low risk of bias.

### 3.3. Meta-Analysis

Most of the articles included in this SR were short-term reports and there were no reports of mortality or cardiovascular events. We extracted and evaluated surrogate outcomes from included articles.

#### 3.3.1. Mono-Block OA_m_ Versus Bi-Block OA_m_

**Figure 5:**
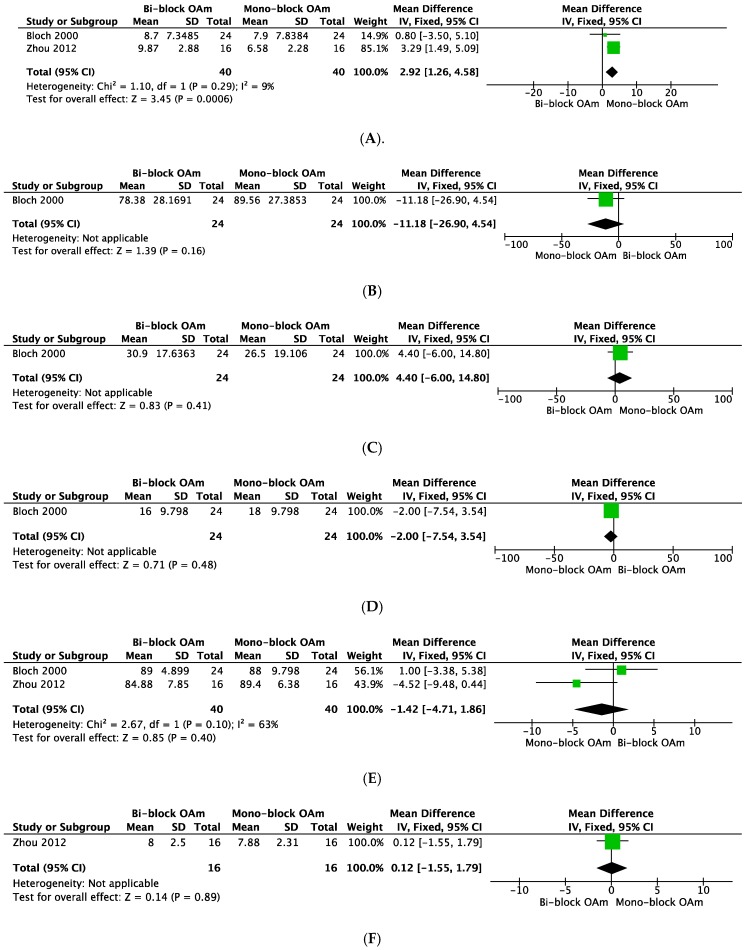

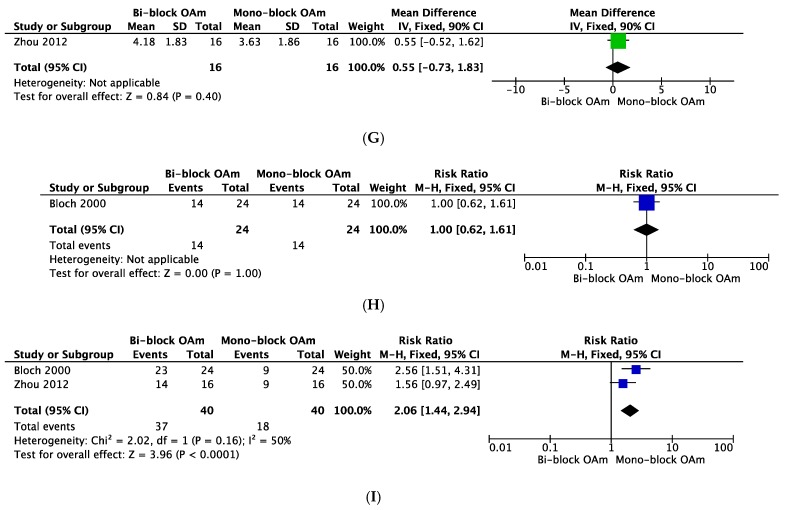
Forest plots of comparisons: Mono-block OA_m_ versus Bi-block OA_m_. Outcomes: (**A**) AHI, (**B**) Lowest SpO_2_, (**C**) Arousal Index, (**D**) Non-REM stage 3, (**E**) Sleep efficiency, (**F**) ESS, (**G**) Snoring Scale, (**H**) Side effects, (**I**) Patient preference. OA_m_: Mandibular advancement type of oral appliance, AHI: Apnea–Hypopnea Index, ESS: Epworth Sleepiness Scale.

The forest plots in the surrogate outcomes (AHI, lowest SpO_2_, arousal Index, NREM stage 3, sleep efficiency, ESS, Snoring Scale, side effects, patient preference) are shown in Figure 5. Comparing Mono-block OA_m_ and Bi-block OA_m_, Mono-block OA_m_ significantly reduced AHI (2.92; 95%CI, 1.26 to 4.58; *p* = 0.0006), and patient preference for Mono-block OA_m_ was significantly higher (2.06; 95%CI, 1.44 to 2.06; *p* < 0.0001). Lowest SpO_2_, arousal index, NREM stage 3, sleep efficiency, ESS, SS, and side effects were not significantly different between the two groups (favoring Mono-block OA_m_, lowest SpO_2_: −11.18; 95%CI, −26.90 to 4.54; *p* = 0.16; arousal index: 4.40; 95%CI, −6.00 to 14.80; *p* = 0.41, NREM stage 3: −2.00; 95%CI, −6.00 to 14.80; *p* = 0.41; sleep efficiency: −1.42, 95%CI, −4.71 to 1.86; *p* = 0.40; ESS: 0.12; 95%CI, −1.55 to 1.79; *p* = 0.89; SS: 0.55; 95%CI, −0.73 to 1.83, *p* = 0.55; side effects: 1.00, 95%CI, 0.62 to 1.61, *p* = 1.00).

#### 3.3.2. Bi-Block OA_m_ Versus Bi-Block OA_m_

Although the study designs adopted in all five articles [17,18,19,20,21] were randomized controlled trials, various OA_m_ devices were utilized, such as Herbst, Thornton Adjustable Positioner (TAP), Klearway, and IST models. Additionally, the material, thickness, and detailed design of each device differed, making it difficult to generate a comprehensive interpretation of results. Therefore, for this systematic review, no meta-analysis was performed regarding these five studies.

### 3.4. GRADE Evidence Profile

Table 3 provides the GRADE evidence profile. A funnel plot was not used to assess publication bias in this meta-analysis because the number of eligible articles was less than 10. As a consequence of risk of bias, inconsistency, indirectness, imprecision, and other considerations, the overall quality of evidence for the AHI and patient preference was rated as low. The quality of evidence for the lowest SpO_2_, arousal index, NREM stage 3, sleep efficiency, ESS, Snoring Scale, and side effects was rated as very low.

## 4. Discussion

In this SR, we investigated which designs were effective when performing OA_m_ treatment for OSA patients. The primary outcome of successful OSA treatment is the improvement of life prognosis and the prevention of cardiovascular disease [22,23]. However, there were no reports of mortality or cardiovascular events in the included articles. The surrogate outcomes are often used, and there are many articles that have reported on surrogate outcomes. Therefore, in this systematic review, we analyzed the surrogate outcomes referred to in previous SRs [6,10,24].

Several systematic reviews on the design of OA_m_ have been reported in the past [25,26,27]. Ahrens et al. [25,26] investigated the subjective patient outcome and polysomnographic indices. They concluded that there was no specific OA_m_ design to improve the subjectively perceived treatment efficiency and the polysomnographic indices effectively. Serra-Torres et al. [27] showed that adjustable and custom-made OA_m_ have a better performance than fixed and prefabricated devices, and that Mono-block OA_m_ cause more adverse effects. However, the application of OA_m_ used in the reports accepted for these systematic reviews did not include the titration of the lower jaw. In this systematic review, we targeted OA_m_ for which titration (including adjustment) of the lower jaw was performed. As for the forward positioning of the lower jaw, many devices are set at 50–75% of the maximum mandibular advancement amount [28,29]. However, a standardized value has yet to be established. Pulling the lower jaw forward expands the upper airway and increases treatment efficacy [30]. However, the burden on the temporomandibular joint, masticatory muscles, and teeth increase, which can lead to an increased risk of side effects such as temporomandibular disorders or tooth movement [31]. In clinical practice, it is necessary to perform the titration of the mandibular position after its initial fixation in order to strike a balance between treatment efficacy and side effects [32]. Therefore, the results of this systematic review can be considered to have high clinical significance. 

There were only two studies comparing Mono-block OA_m_ and Bi-block OA_m_. Meta-analysis results showed that Mono-block OA_m_ were more effective in terms of AHI and patient preference values. During sleep, humans perform various jaw movements such as sleep bruxism and swallowing; however, for most of the time during sleep, the mouth remains open [33]. Generally, skeletal muscles relax and muscle tonus decreases during sleep [34]. The same phenomenon is observed for masticatory muscles, and it is assumed that the opening of the mouth is a result of the relaxation of muscles responsible for keeping the mouth closed [35]. It is assumed that in some cases, the tongue and soft palate retract with the opening of the mouth, which leads to a narrowing of the respiratory tract and resulting respiratory problems. Bi-block OA_m_ contain various adjustment mechanisms to easily adjust the position of the lower jaw. Such layouts make it possible to open the mouth more or less freely. However, this also implies an increased chance of the lower jaw moving backward and downward during sleep. That is to say, the improvement of AHI values may be poor. On the other hand, with Mono-block OA_m_, the upper and lower jaws are fixed in one position and mouth movement is often restricted. Therefore, as also reported by Bloch et al. [15] and Zhou et al. [16], AHI values improved at a better rate with Mono-block OA_m_ compared to Bi-block OA_m_. 

There was also a report claiming that AHI values decreased significantly with Bi-block OA_m_. It should be noted that the average BMI and AHI values differed from case to case. Ghazal et al. [20] reported a small decrease in AHI from 21.5 ± 13.5 to 11.1 ± 11.8 (48.4% decrease) after using IST devices for six months (*n* = 47). However, after 24 months (*n* = 24), AHI values significantly improved, from 18.4 ± 8.9 to 4.6 ± 5.8 (75.0% decrease). Further, after six months of use of TAP devices for control (*n* = 48), AHI values significantly improved from 19.8 ± 12.7 to 6.7 ± 9.1 (68.8% decrease). After 24 months (*n* = 21), the device remained effective and AHI values improved significantly from 19.8 ± 12.7 to 5.4 ± 5.1 (72.7% decrease). There are various types of Bi-block OA_m_; however, a collective opinion as to which design is most effective has not yet been formed. This systematic review only includes studies in which titration or post-adjustment comparison was performed. Regarding the AHI results, it was inferred that a large amount of mandibular advancement was set in the Mono-block OA_m_ with complicated adjustment. Additionally, the capability of opening the mouth granted by Bi-block OA_m_ was also considered to be a contributing factor. Further, the ESS, Snoring Scale, and side effects meta-analysis results showed no difference between Mono-block and Bi-block OA_m_. Although no detailed descriptions concerning adherence were given, no differences were found. However, it should be noted that the patient preference values were higher for Mono-block OA_m_. According to Bloch et al. [15], 15 out of 24 subjects (62.5%) preferred Mono-block OA_m_, whereas one subject (6.7%) preferred the Herbst model, which is a Bi-block OA_m_. The following reasons for the preference of Mono-block OA_m_ were reported: greater alleviation of OSA symptoms (*n* = 11), robustness and ease of installation (*n* = 5), and less side effects (*n* = 4). The subject who preferred the Herbst model stated an impression of greater alleviation of OSA symptoms. Further, according to Zhou et al. [16], seven out of 16 subjects (43.8%) preferred Mono-block OA_m_, whereas two subjects (12.5%) preferred the SILENT NITE model, which is a Bi-block OA_m_. Although detailed reasons regarding preferences for each device were not clear, it was reported that six subjects experienced equipment failure with the SILENT NITE model during the study period, which may have contributed to the high number of subjects preferring Mono-block OA_m_. It can be said that devices producing high patient preference values seem to satisfy not only in terms of therapeutic efficacy and side effect alleviation, but also the ease of installation and durability of the OA_m_. Although Mono-block OA_m_ evoke an image of severe constraint, this sense of constraint does not necessarily seem to be reflected in the patient preference values. In recently reported cases of OSA patients with locomotive syndrome and paralysis after a cerebral infarction [36], methods that allow for an easy installation seem to be preferred. In particular, Mono-block devices utilizing soft resin can be installed with only one hand. It seems that the choice of equipment also has to be considered in the context of each individual patient. 

It should be noted that our systematic review was conducted under the following limitations. Firstly, a small sample size and limited number of studies were included in the review, with many reports covering only short research periods. Secondly, we included studies with a high risk of bias due to subject blind testing or incomplete data. Follow-up research will require a revised research design and greater sample size. In particular, OA_m_ treatment is highly symptomatic and conducted over a long period of time, which necessitates further investigations of adherence and side effects.

## 5. Conclusions

Despite frequently small sample sizes and a majority of short-term reports, current evidence shows that Mono-block OA_m_ are more effective than Bi-block OA_m_ for OSA patients. Further well-designed, larger trials are required to determine the benefit for patients.

## Figures and Tables

**Table 1:** Characteristics of the included studies for systematic review.

Author	Study Design	Subjects	Interventions	Age	BMI	Follow-up	Outcomes
Mono-block OA_m_ vs Bi-block OA_m_							
Bloch 2000	Cross over- RCT	24	Mono-block	50.6 ± 1.5 ^a^	27.4 ± 0.8 ^a^	(156 ± 14 days) + 1 week	AHI, ESS, Snoring (VAS), QOL, Side effect, Adherence, Patients preference
			Herbst				
Zhou 2012	Cross over- RCT	16	Mono-block	45.23 (26.3–55.4) ^b^	26.67 (22.3–29.8 ) ^b^	3 months	AHI, ESS, Snoring Scale, Airway space, Side effect, Adherence, Patient preference
			SILENT NITE				
Bi-block OA_m_ vs Bi-block OA_m_							
Lawton 2005	Cross over- RCT	16	Herbst	44.8 (24.0–68.4) ^c^	29.2 (23.8–51.1) ^c^	4–6 weeks	AHI, LowestspO2, ESS, Sleepiness (VAS), Snoring (VAS), SF-36, Side effects
			Twin Block				
Bishop 2014	Cross over- RCT	18	TAP	47.6 ± 2.6 ^a^	31.4 ± 1.0 ^a^	1–2 months	RDI, ESS, SAQLI, Patient preference
			Klearway				
Ghazal 2009	RCT	103	IST	55.5 ± 10.6 ^d^	25.9 ± 2.9 ^d^	6 months, 24 months	AHI, LowestspO2, Arousal Index, Sleep stage, Sleep efficiency, ESS, PSQI, SF-36, Adherence, Patient preference
			TAP				
Rose 2002	Cross over- RCT	26	Silencer	56.8 ± 5.2 ^d^	27.5 ± 3.1 ^d^	6–8 weeks	RDI, LowestspO2, Sleepiness (VAS), Sleep quality (VAS), Snoring (VAS), Adherence
			Karwetzky activator				
Gautheir 2009	Cross over- RCT	16	Silencer	56.8 ± 5.3 ^a^	25.9 ± 2.1 ^a^	3 months	RDI, LowestspO2, Sleep stage, Sleep efficiency, Blood pressure, ESS, FSS, FOSQ, Patient preference
			Klearway				

OA_m_: Mandibular advancement type of oral appliance, AHI: Apnea-Hypopnia Index, ESS: Epworth Sleepiness Scale. RDI: Respiratory disturbance index, SAQLI: Sleep Apnea Quality of Life Index, PSQI: Pittsburgh Sleep Quality Index. FSS: Fatigue Severity Scale, FOSQ: Functional Outcomes of Sleep Questionnaire. ^a^ mean ± SE, mean (SE). ^b^ mean (minimum, maximum). ^c^ median (quartile range). ^d^ mean ± SD.

**Table 2:** Continued.

Study	Interventions	Follow Up	AHI (RDI)			Key Results	Conclusion
			Baseline	wth OA_m_	%Reuction		
Mono-block OA_m_ vs Bi-blockOA_m_							
Bloch 2000	Mono-block	156 ± 14 days	22.6 ± 3.1 ^a^	7.9 ± 1.6 ^a^	65.0	Fifteen patients preferred the Monobloc, eight patients had no preference, and one patient preferred the Herbst device	Both the Herbst and the Mono-bloc are effective therapeutic devices for sleep apnea. The Mono-bloc relieved symptoms to a greater extent than the OSA-Herbst, and was preferred by the majority of patients on the basis of its simple application.
	Herbst		22.6 ± 3.1 ^a^	8.7 ± 1.5 ^a^	61.5		
Zhou 2012	Mono-block	3 months	26.38 ± 4.13 ^b^	6.58 ± 2.28 ^b^*	75.1	The monoblock OA_m_ was statistically more efficient in reducing AHI and Apnoea Index (AI) than the SILENT NITE (*p* < 0.05)	The Both OA_m_ showed good efficacy in the treatment for mild to moderate OSA. Use of the monoblock appliance should be considered when patients with OSA choose OA treatment, as it was more efficient in reducing the AHI compared to the two-piece appliance and was preferred by most patients.
	SILENT NITE		26.38 ± 4.13 ^b^	9.87 ± 2.88 ^b^*	62.6		
Bi-block OA_m_ vs Bi-block OA_m_							
Lawton 2005	Twin Block	4–6 weeks	45.5 (29.0–68.0) ^c^	34.0 (9.0–63.0) ^c^	25.3	The Herbst OA_m_ proved to be the more effective appliance for reducing daytime sleepiness (*p* = 0.04) and was the more popular appliance among the patients.	The Twin Block OA_m_ represents a viable alternative to the Herbst OA_m_ in the treatment of patients with OSA.
	Herbst		45.5 (29.0–68.0) ^c^	24.5 (0.0–45.0) ^c^	46.2		
Bishop 2014	TAP	1–2 months	16.5 ± 3.2 ^a^	7.7 ± 3.3 ^a^	53.3	There were no significant statistical differences in treatment outcomes between the two appliances. There was a statistically significant (*p* < 0.05) preference for a OA_m_ design with minimal coverage of teeth and palate.	There was a trend toward greater improvement with the appliance with less acrylic resin bulk and less interocclusal contact. OA selection should favor titratable, unobtrusive designs with appropriate construction to promote acceptance and adherence to OA therapy.
	Klearway		16.5 ± 3.2 ^a^	10.3 ± 3.2 ^a^	37.6		
Ghazal 2009	IST	6 months	21.5 ± 13.5 ^b^	11.1 ± 11.8 ^b^*	48.4	Quality of life, sleep quality, sleepiness, symptoms and sleep outcome showed significant improvement in the short-term evaluation with both appliances, but the TAP revealed a significantly greater effect. After more than 2 years of treatment, sleep outcomes revealed an equal effect with both appliances.	This study illustrates that both the IST and the TAP appliances are effective therapeutic devices for OSA after a period of over 24 months.
	TAP		21.5 ± 16.9 ^b^	6.7 ± 9.1 ^b^*	68.8		
	IST	24 months	18.4 ± 8.9 ^b^	4.6 ± 5.8 ^b^	75.0		
	TAP		19.8 ± 12.7 ^b^	5.4 ± 5.1 ^b^	72.7		
Rose 2002	Silencer	6–8 weeks	16.0 (4.4) ^b^	7.4 (5.3) ^b^*	53.8	The results showed that a statistically significant improvement in the respiratory parameters was achieved with both appliances (*p* < 0.01). However, the activator was significantly more effective (*p* < 0.01) than the Silencor.	Both appliances reduced daytime sleepiness and snoring and improved sleep quality, and both influenced the treatment outcome.
	Karwetzky activator		16.2 (4.6) ^b^	5.5 (3.3) ^b^*	66.0		
Gautheir 2009	Silencer	3 months	10.0 ± 1.2 ^a^	4.7 ± 0.9 ^a^*	53.0	The RDI was slightly lower with the Silencer (*p* < 0.05) but subjects’ preference for comfort was in favor of the Klearway (*p* = 0.04).	Although both OA_m_ decreased RDI and subjective daytime sleepiness in a similar manner, the choice between various types of OA_m_ needs to be taken into account when considering the benefit of RDI reduction over the benefit of subject compliance.
	Klearway		10.0 ± 1.2 ^a^	6.5 ± 1.3 ^a*^	35.0		

OA_m_: Mandibular advancement type of oral appliance, AHI: Apnea hyapopnea index. ^a^ mean ± SE, means (SE). ^b^ mean ± SD. ^c^ median (quartile range). * *p* < 0.05: the comparion of two OA_m_.

**Table 3:** GRADE evidence profile in the comparison of Mono-block OAm and Bi-block OAm.

Certainty Assessment							No. of Patients		Effect		Certainty
No. of Studies	Study Design	Risk of Bias	Inconsistency	Indirectness	Imprecision	Other Considerations	Mono-block OA_m_	Bi-block OA_m_	Relative	Absolute	
									(95% CI)	(95% CI)	
AHI											
2	randomised trials	serious ^a^	not serious	not serious	serious ^b^	none	40	40	-	MD 2.92 higher	LOW
										(1.26 higher to 4.58 higher)	
Lowest SpO2											
1	randomised trials	serious ^a^	not serious	not serious	very serious ^b,c^	none	24	24	-	MD 11.18 lower	VERY LOW
										(26.9 lower to 4.54 higher)	
Arousal Index											
1	randomised trials	serious ^a^	not serious	not serious	very serious ^b,c^	none	24	24	-	MD 4.4 higher	VERY LOW
										(6 lower to 14.8 higher)	
Non-REM Stage 3											
1	randomised trials	serious ^a^	not serious	not serious	very serious ^b,c^	none	24	24	-	MD 2 lower	VERY LOW
										(7.54 lower to 3.54 higher)	
Sleep Efficiency											
2	randomised trials	serious ^a^	serious ^d^	not serious	very serious ^b,c^	none	40	40	-	MD 1.42 lower	VERY LOW
										(4.71 lower to 1.86 higher)	
ESS											
1	randomised trials	serious ^a^	not serious	not serious	very serious ^b,c^	none	16	16	-	MD 0.12 higher	VERY LOW
										(1.55 lower to 1.79 higher)	
Snoring Scale											
1	randomised trials	serious ^a^	not serious	not serious	very serious ^b,c^	none	16	16	-	MD 0.55 higher	VERY LOW
										(0.73 lower to 1.83 higher)	
Side effect											
1	randomised trials	serious ^a^	not serious	not serious	very serious ^b,c^	none	14/24 (58.3%)	14/24 (58.3%)	RR 1.00	0 fewer per 1000	VERY LOW
									(0.62 to 1.61)	(from 222 fewer to 356 more)	
Patient preference											
2	randomised trials	serious ^a^	not serious ^e^	not serious	serious ^b^	none	37/40 (92.5%)	18/40 (45.0%)	RR 2.06	477 more per 1000	LOW
									(1.44 to 2.94)	(from 198 more to 873 more)	

CI: Confidence interval, MD: Mean difference, RR: Risk ratio, AHI: Apnea Hypopnea Index, ESS: Epworth Sleepiness Scale. a. The risk of bias in included studies were high. b. The number of patients were very small. c. 95% CI contained no effect. d. Heterogeneity: I^2^ = 63%. e. Heterogeneity: I^2^ = 50%, but the direction of effect in two studies was same. f. Heterogeneity: I^2^ = 66%.
